# Roles of TET and TDG in DNA demethylation in proliferating and non-proliferating immune cells

**DOI:** 10.1186/s13059-021-02384-1

**Published:** 2021-06-22

**Authors:** Atsushi Onodera, Edahí González-Avalos, Chan-Wang Jerry Lio, Romain O. Georges, Alfonso Bellacosa, Toshinori Nakayama, Anjana Rao

**Affiliations:** 1 Division of Signaling and Gene Expression, La Jolla Institute for Immunology, 9420 Athena Circle, La Jolla, CA 92037 USA; 2 Department of Immunology, Graduate School of Medicine, Chiba University, 1-8-1 Inohana, Chuo-ku, Chiba, 260-8670 Japan; 3 Institute for Global Prominent Research, Chiba University, 1-33, Yayoicho, Inage-ku, Chiba, 263-8522 Japan; 4 Bioinformatics and Systems Biology Graduate Program, University of California, San Diego, 9500 Gilman Drive, La Jolla, CA 92093 USA; 5 Present address: Department of Microbial Infection and Immunity, Ohio State University, 460 W 12th Ave, Columbus, OH 43210 USA; 6 Cancer Signaling and Epigenetics Program & Cancer Epigenetics Institute, Fox Chase Cancer Center, 333 Cottman Avenue, Philadelphia, PA 19111 USA; 7 AMED-CREST, AMED, 1-8-1 Inohana, Chuo-ku, Chiba, 260-8670 Japan; 8 Department of Pharmacology and Moores Cancer Center, University of California, San Diego, 9500 Gilman Drive, La Jolla, CA 92093 USA; 9 Sanford Consortium for Regenerative Medicine, 2880 Torrey Pines Scenic Drive, La Jolla, CA 92037 USA

## Abstract

**Background:**

TET enzymes mediate DNA demethylation by oxidizing 5-methylcytosine (5mC) in DNA to 5-hydroxymethylcytosine (5hmC), 5-formylcytosine (5fC), and 5-carboxylcytosine (5caC). Since these oxidized methylcytosines (oxi-mCs) are not recognized by the maintenance methyltransferase DNMT1, DNA demethylation can occur through “passive,” replication-dependent dilution when cells divide. A distinct, replication-independent (“active”) mechanism of DNA demethylation involves excision of 5fC and 5caC by the DNA repair enzyme thymine DNA glycosylase (TDG), followed by base excision repair.

**Results:**

Here by analyzing inducible gene-disrupted mice, we show that DNA demethylation during primary T cell differentiation occurs mainly through passive replication-dependent dilution of all three oxi-mCs, with only a negligible contribution from TDG. In addition, by pyridine borane sequencing (PB-seq), a simple recently developed method that directly maps 5fC/5caC at single-base resolution, we detect the accumulation of 5fC/5caC in TDG-deleted T cells. We also quantify the occurrence of concordant demethylation within and near enhancer regions in the *Il4* locus. In an independent system that does not involve cell division, macrophages treated with liposaccharide accumulate 5hmC at enhancers and show altered gene expression without DNA demethylation; loss of TET enzymes disrupts gene expression, but loss of TDG has no effect. We also observe that mice with long-term (1 year) deletion of *Tdg* are healthy and show normal survival and hematopoiesis.

**Conclusions:**

We have quantified the relative contributions of TET and TDG to cell differentiation and DNA demethylation at representative loci in proliferating T cells. We find that TET enzymes regulate T cell differentiation and DNA demethylation primarily through passive dilution of oxi-mCs. In contrast, while we observe a low level of active, replication-independent DNA demethylation mediated by TDG, this process does not appear to be essential for immune cell activation or differentiation.

**Supplementary Information:**

The online version contains supplementary material available at 10.1186/s13059-021-02384-1.

## Background

DNA cytosine methylation is the classic “epigenetic” mark. It is controlled by the functional interplay between two families of enzymes: DNA methyltransferases (DNMTs) and TET methylcytosine dioxygenases, which control DNA methylation and demethylation, respectively [1–4]. DNMTs transfer a methyl group from S-adenosylmethionine (SAM) to the 5 position of cytosine to generate 5-methylcytosine (5mC), the bulk of which is present in the CpG (CG) sequence context [1–5], whereas TET proteins are Fe(II) and 2-oxoglutarate-dependent dioxygenases that oxidize the methyl group of 5-methylcytosine (5mC) to 5-hydroxymethylcytosine (5hmC), 5-formylcytosine (5fC), and 5-carboxylcytosine (5caC) in DNA [6–9]. TET enzymes are now known to mediate essentially all of the dynamic DNA demethylation that occurs in mammalian genomes during embryogenesis, cell lineage specification in developing embryos and organs, and cell differentiation in response to environmental cues [3, 10, 11].

There are at least two mechanisms by which TET proteins can mediate DNA demethylation. The first, known as “passive” or replication-dependent DNA demethylation, occurs during DNA replication, when 5mC complementary to the G in CG sequences in the template strand is replaced with unmodified cytosine (C) in the newly synthesized DNA strand. The resulting hemi-methylated CpG sequences are rapidly remethylated by the maintenance methyltransferase complex of DNMT1 and UHRF1 [12, 13]. UHRF1 recognizes hemi-methylated CpGs through its SRA domain [1, 14, 15], and the DNMT1/UHRF1 complex interacts with proliferating cell nuclear antigen (PCNA) and travels with the DNA replication machinery [14, 15]. This process restores symmetrical DNA methylation to newly synthesized DNA and is responsible for the partial heritability of DNA methylation. However, the DNMT1/UHRF1 complex does not recognize hemi-modified CpGs that contain 5hmC, 5fC, or 5caC, which thus progressively lose DNA methylation as a function of DNA replication [16, 17]. The second mechanism, sometimes known as “active” or replication-independent DNA demethylation, involves the DNA repair enzyme thymine DNA glycosylase (TDG), which, in addition to its function of excising thymine from mismatched T:G base pairs in DNA, can also efficiently excise 5fC and 5caC from correctly base-paired 5fC:G and 5caC:G [6, 18, 19]. The resulting abasic sites are subject to base excision repair, leading to DNA demethylation via replacement of the original 5fC or 5caC with unmodified C [20].

Here we examine the respective roles of TET and TDG in T cell and macrophage differentiation using very deep sequencing to determine DNA methylation status at selected gene loci. As an example of a process that involves many cycles of DNA replication, we focused on *Il4* gene expression by differentiating Th2 cells, a system we had investigated earlier with cruder tools [21]. We show that acute inducible deletion of all three *TET* genes diminishes Th2 differentiation while increasing DNA methylation at numerous enhancers, whereas similarly acute, inducible deletion of *Tdg* had no effect on either DNA demethylation or Th2 differentiation. We also document concordant demethylation near enhancer regions in the *Il4* locus, confirm the importance of GATA3 for DNA demethylation during Th2 differentiation, and identify at least one region whose demethylation does not appear to be dependent on either TET or TDG. Overall, we conclude that demethylation during Th2 differentiation is mainly due to passive DNA demethylation of all three oxidized methylcytosines, with only a very minor contribution (if any) of replication-independent DNA demethylation via TDG.

To assess DNA methylation changes during cell differentiation in the absence of DNA replication, we chose to examine bone marrow-derived macrophages stimulated by treatment with liposaccharide (LPS) for 6 h. These cells undergo cell cycle arrest, do not enter S phase, and stop proliferating in this time frame, but show considerable alterations in gene expression and activate numerous enhancers [22]. Deletion of all three *TET* genes altered gene expression and increased 5hmC deposition at selected enhancers in differentiating macrophages, without altering DNA methylation as assessed by bisulfite sequencing (BS-seq), whereas loss of TDG had no effect either on gene expression or on DNA methylation status at the enhancers, although the enhancers accumulated low levels of 5fC/5caC. Moreover, mice remained healthy after acute tamoxifen-mediated deletion of *Tdg*, with essentially normal hematopoiesis for more than 1 year. We conclude that LPS-treated macrophages accumulate 5hmC at certain enhancers and increase the expression of associated genes without DNA demethylation and that TDG has no effect on either gene expression or DNA demethylation in these cells.

## Results

### TET deficiency impairs Th2 differentiation and IL-4 production but TDG deficiency has little effect

**Fig. 1:**
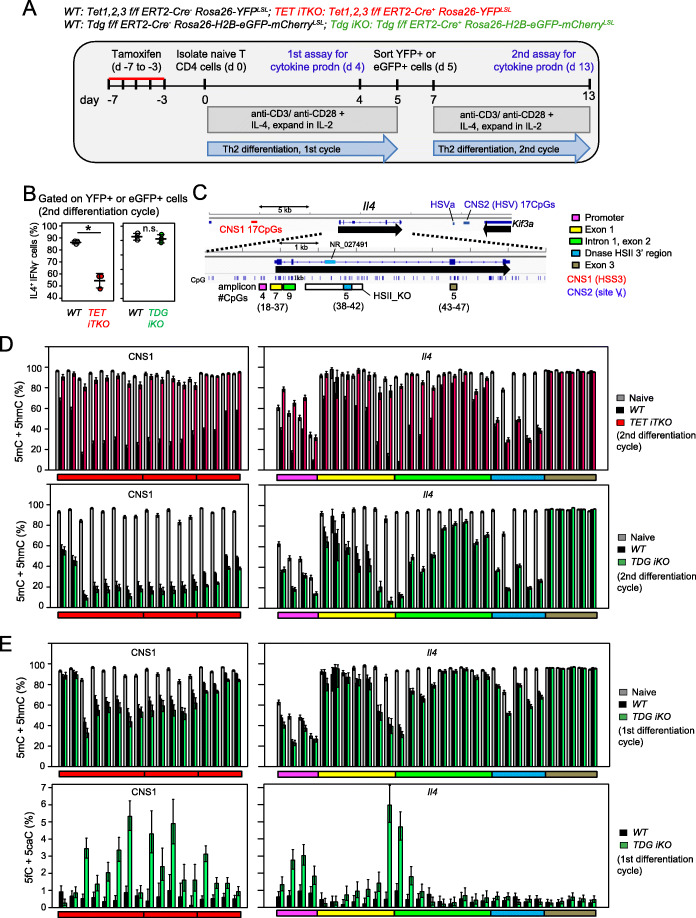
TET enzymes are important, but TDG is dispensable, for IL-4 production and DNA demethylation of the *Il4* locus. **A** Flowchart of experiments. **B** Quantification of IL-4 production by Th2 cells after the second cycle of differentiation of naïve T cells from *WT* vs. *TET iTKO*, or *WT* vs. *TDG iKO* mice. **C** Schematic representation of the *Il4* locus. The locations of all CpGs, the locations of PCR amplicons, and the numbers of CpGs per amplicon are indicated. **D** Bar graphs show the percentage of (5mC + 5hmC)/total C in 47 CpGs in the *Il4* locus with confidence intervals (CIs) in *WT* vs. *TET iTKO* cells (upper), or *WT* vs. *TDG iKO* cells (lower), as determined by BS-seq. Results for naïve CD4^+^ T cells and Th2 cells after the second cycle of differentiation are shown. Data are representative of two independent experiments. Note that the *CNS1* enhancer and the intronic enhancer near the border of exon 1 and intron 1 undergo substantial demethylation during Th2 differentiation. **E** Bar graphs show the percentages of (5mC + 5hmC)/total C (upper) and (5fC + 5caC)/total C (lower) in 47 CpGs in the *Il4* locus in *WT* vs. *TDG iKO* cells, as determined by BS-seq (upper) and PB-seq (lower). Results for naïve CD4^+^ T cells (adapted from **D**) and Th2 cells after a single cycle of differentiation are shown. Data are representative of two independent experiments. TDG deficiency results in clear increases in 5fC/5caC, but no decrease in 5mC+5hmC, at CpGs that undergo TET-dependent demethylation. Statistical significance was calculated using an unpaired two-tailed t test. *P < 0.05

To assess whether deletion of TETs or TDG could affect IL-4 production in Th2 cells, we used *Tet1*^*fl/fl*^ *Tet2*^*fl/fl*^ *Tet3*^*fl/fl*^ *Rosa26-YFP*^*LSL*^ (*Tet1/2/3*^*fl/lf*^) *ERT2-Cre* or *Tdg*^*fl/fl*^ *ERT2-Cre Rosa26-H2B-EGFP/GPI-mCherry*^*LSL*^, in which YFP and GFP serve as reporters of Cre expression respectively (Fig. 1A; Additional file 1: Fig. S1). TET or TDG deletion can be induced efficiently in these mice (termed *TET iTKO* and *TDG iKO* mice, respectively, where *i* indicates inducible) by treating them with tamoxifen for 5 days [23, 24]. Naïve CD4^+^ T cells were differentiated under Th2 conditions for one or two cycles and then restimulated for 4 h with PMA and ionomycin, and production of the cytokines IL-4 and IFN-γ was measured by intracellular staining and flow cytometry. *Tet1/2/3*-deleted cells produced considerably less IL-4 than control T cells, both in the first cycle of differentiation and after reactivation and culture for a second week, which induced a further increase in the frequency of control IL-4-expressing cells from ~60% to nearly 90% (Fig. 1B; Additional file 1: Fig. S1A-C). In contrast, *Tdg* deletion had little effect on IL-4 production after either 1 or 2 weeks of Th2 differentiation (Fig. 1B, *right*;Additional file 1: Fig. S1D-F). GATA3 protein expression levels were not affected by TET or TDG deletion (Additional file 1: Fig. S1G, H). We repeated these experiments for a different direction of naïve T cell differentiation, the generation of “induced” T regulatory (iTreg) cells by activation of naïve T cells in the presence of TGF-β (Additional file 1: Fig. S1I). We previously showed that *Tet2/Tet3* gene deletion compromises gene expression, DNA methylation patterns, and functional activities of these cells [24, 25]. In contrast, *Tdg* deletion was dispensable for Foxp3 expression by iTregs (Additional file 1: Fig. S1J, K), as well as the maintenance of Foxp3 expression after restimulation (Additional file 1: Fig. S1L, M). Thus, expression of both Foxp3 and IL-4, by differentiating iTregs and Th2 cells, respectively, requires TET but not TDG.

IL-4 production during differentiation to Th2 cells depends on cell division [26], which facilitates “passive,” replication-dependent demethylation by dilution of oxi-mC bases in DNA [16, 17]. To ask if the decrease in IL-4 production by Tet1/2/3-deficient Th2 cells occurred early or after multiple cycles of cell division, we labeled naïve T cells with Cell Trace Violet (a dye which is diluted by half in daughter cells produced after each round of cell division), then stimulated the cells and assessed their production of IL-4 and IFN-γ as a function of cell division by gating on cells that had divided 0, 1, 2, 3 or 4 times. The decrease in IL-4 production as a result of TET deficiency was apparent even at the first cell division, when most fully methylated DNA in naïve T cells becomes hemi-modified; the decrease became more prominent thereafter, as the fraction of DNA that is fully demethylated on both strands increases to 50% at 2 cell divisions, 75% at 3 divisions, and so on (Additional file 1: Fig. S1N). These results are consistent with our previous conclusion [21] that DNA demethylation occurs in a “passive,” replication-dependent manner during Th2 differentiation, with the highest degree of DNA demethylation observed in cells transcribing the highest levels of IL-4. This is a general phenomenon: whole-genome bisulfite sequencing (WGBS) of a variety of different cell types shows that DNA demethylation is most pronounced at promoters of the most highly transcribed genes, and extends most strongly into the gene body of these genes [27, 28]. We note that most DNA demethylation is driven by transcription factors and is a consequence, not a cause, of high gene transcription [29, 30].

### TET deficiency impairs DNA demethylation at the *Il4* locus but TDG deficiency has little effect

To examine the effects of TET and TDG deficiency in Th2 cells on DNA cytosine modification, we performed bisulfite (BS) sequencing of specific CpG-containing amplicons spanning known regulatory regions of the *Il4* gene. In preference to performing WGBS, which requires very deep sequencing (>500 million reads) to obtain adequate coverage of a sufficient number of CpGs across the genome, we chose to focus on a single gene locus and perform high-coverage amplicon sequencing, so as to assure very high sequence coverage of all CpGs within the regions of interest. We assessed DNA modification (5mC+5hmC) at two distant regulatory regions (conserved non-coding sequences, CNS), *CNS1* (HSS3) and *CNS2* (site V), located 5′ and 3′ of the *Il4* gene respectively [31–33]; the *Il4* promoter, exon 1 and intron 1; the 3′ region of the conserved enhancer HSII; and exon 3. Together, these seven amplicons cover 64 CpG dinucleotides across the *Il4* locus (Fig. 1C). Although BS-seq measures the sum of 5mC and 5hmC [34], 5hmC is <10% of 5mC in WT naïve T cells, drops to very low levels in differentiated T cells, and is absent in TET-deficient cells [24, 35, 36], leading us to refer sometimes to the results of bisulfite sequencing simply as “methylation” for convenience.

**Fig. 2:**
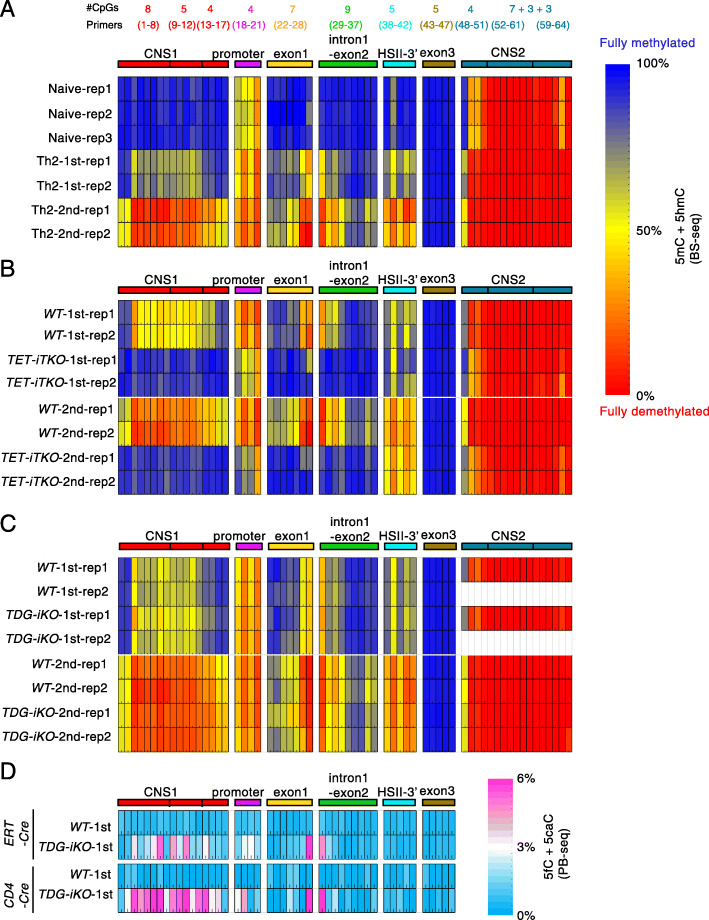
Summary of experiments showing TET-dependent, TDG-independent demethylation of the *Il4* locus during Th2 differentiation. **A** Heat maps depicting the percentage of (5mC + 5hmC)/total C in 64 CpGs in the *Il4* locus in wildtype naïve CD4^+^ T cells and Th2 cells after the first and second cycles of differentiation, as determined by BS-seq. Data show the results of thousands of sequencing reads from two or three independent experiments. The numbers of CpGs in each amplicon are shown above the heat maps. **B**, **C** Heat maps depicting the percentage of (5mC + 5hmC)/total C in 64 CpGs in the *Il4* locus in *WT* vs. *TET iTKO* cells (**B**), or *WT* vs. *TDG iKO* cells (**C**), as determined by BS-seq. Results for Th2 cells after the first and second cycles of differentiation are shown. The data show the results of thousands of sequencing reads from two independent experiments. **D** Heat maps depicting the percentage of (5fC + 5caC)/total C in 47 CpGs in the *Il4* locus in *WT* vs. *TDG iKO* cells, as determined by PB-seq. *Tdg* gene deletion was achieved using *Cre-ERT2* (*upper*) or *CD4Cre* (*lower*). Results for Th2 cells after the first cycle of differentiation are shown

Figures 1D and E show bar graphs of representative amplicon BS-seq data for the *Il4* gene (for heat map with summary of all data, see Fig. 2). All CpGs in *CNS1*, exon 1, intron 1, exon 2, and the 3′ region of HSII were essentially fully methylated (5mC+5hmC) in WT naive CD4^+^ T cells (Fig. 1D, *gray bars*) and became substantially demethylated during Th2 differentiation (Fig. 1D, *black bars*); also see Fig. 2A. As previously reported [21], the promoter region and most CpGs in *CNS2* were poorly methylated (5mC+ 5hmC) in naive T cells and remained demethylated in differentiated Th2 cells (Fig. 2A; Additional file 1: Fig. S1O), whereas exon 3, which was heavily methylated, exhibited no change in methylation levels even after two cycles of cultivation (Figs. 1D and 2A). These data are consistent with our previous findings, obtained by digestion of genomic DNA of naïve and Th2 cells with the restriction enzyme McrBC, which preferentially cleaves heavily methylated DNA [21].

We examined the effects of *Tet1/2/3* and *Tdg* deletion on the modification status of the *Il4* gene during Th2 differentiation. WT Th2 cells showed a substantial decrease in 5mC + 5hmC at *CNS1*, exon 1, intron 1, and exon 2 as described above, whereas *Tet1/2/3 iTKO* cells did not (Fig. 1D, *top*, *compare black and red bars*; Fig. 2B). In contrast, WT and *TDG iKO* cells showed a similar decrease of 5mC and 5hmC during both the first (Fig. 1E, *top*) and second (Fig. 1D, *bottom; compare black and green bars*; Fig.  2C) cycles of differentiation. Notably, one region of the *Il4* locus—the 3′ region of HSII (CpGs 38-42)—became demethylated in a manner apparently independent of both TET (Fig. 1D, *top*) and TDG (Fig. 1D, *bottom*). Thus, triple TET deficiency substantially impaired Th2 differentiation and almost completely eliminated the demethylation observed in *Il4* regulatory regions in the differentiating cells, whereas TDG deficiency affected neither Th2 differentiation nor demethylation of the *Il4* locus during Th2 differentiation.

Since DNA demethylation in the *Il4* locus during Th2 differentiation was dependent on DNA replication, we asked whether the increased DNA methylation in TET-deficient cells reflected an increase in the levels of the maintenance DNA methyltransferase DNMT1 and its obligate protein partner UHRF1. Unexpectedly, we found that *TET iTKO* cells showed a clear decrease in both DNMT1 and UHRF1 protein levels compared to WT Th2 cells; in contrast, there was no perceptible change in either DNMT1 or UHRF1 protein levels in WT compared to *TDG iKO* Th2 cells (Additional file 1: Fig. S1P, Q). The decrease of DNMT1 and UHRF1 proteins in *TET iTKO* Th2 cells might in part account for the observed demethylation at the 3′ region of HSII in these cells (also see the “Discussion” section).

### TDG deficiency results in low-level 5fC/5caC accumulation with no change in overall gene expression

To confirm that TDG indeed excised 5fC and 5caC, we treated Th2 cells from the 1st differentiation cycle with pyridine borane (PB), which converts 5fC and 5caC into dihydrouracil (DHU) [37, 38]. TET-assisted pyridine borane sequencing (TAPS) was originally developed for bisulfite-free detection of 5mC and 5hmC [37], but we applied it here for single-base resolution mapping of 5fC and 5caC. We observed a substantial accumulation of 5fC/5caC (4–6% of C) at the *Il4* gene locus in *TDG iKO* Th2 cells compared to WT (Fig. 1E, *bottom*; Fig. 2D). The 5fC/5caC accumulation was observed only in regions such as *CNS1*, exon 1, and intron 1 which undergo TET-dependent DNA demethylation (Fig. 1E, *bottom*; Fig. 2D), but not at regions such as exon 3, which do not undergo DNA demethylation during Th2 differentiation (Fig. 1E, *bottom right*; Fig. 2D), confirming that TDG excised 5fC and 5caC generated by TET proteins.

Together, these results confirm that in addition to its known activity of excising thymine from T:G mismatches, TDG can also excise TET-generated 5fC and 5caC from correctly base-paired CpG:GpC sequences in DNA, as also shown previously for other cell types [39, 40]. However, TDG deletion did not result in major changes in gene expression assessed by RNA-seq (Additional file 1: Fig. S2). We conclude that under standard conditions of Th2 differentiation, TDG is not essential for *Il4* locus demethylation, *Il4* gene expression, IL-4 cytokine production, or overall patterns of gene expression; moreover, the presence of TDG in normal naive T cells does not accelerate the process of Th2 differentiation.

### TET deficiency alters gene expression in non-proliferating macrophages but TDG deficiency has little effect

**Fig. 3:**
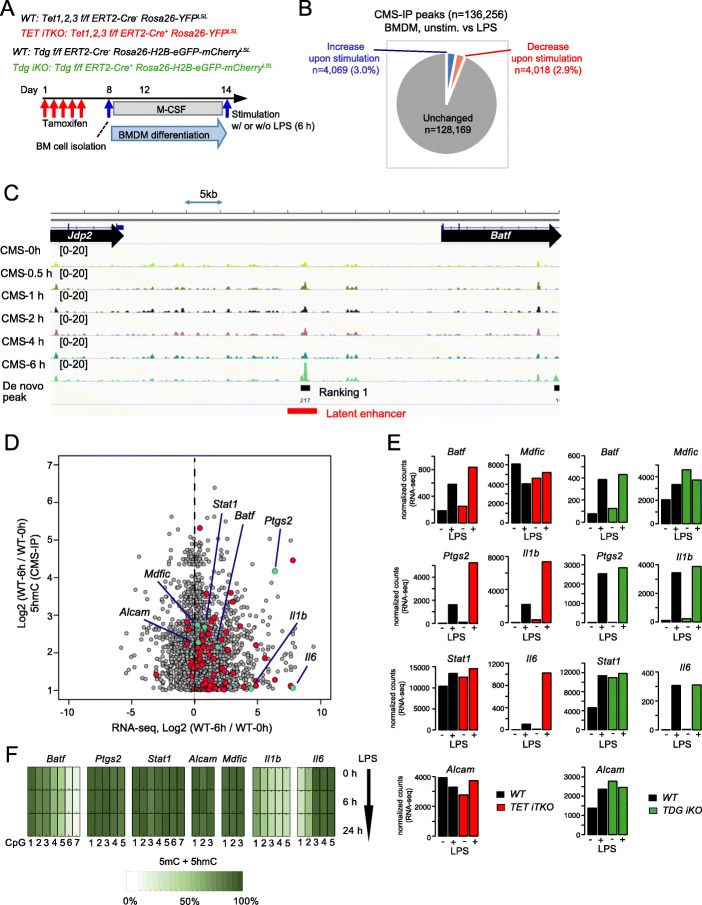
LPS stimulation of bone marrow-derived macrophages induces 5hmC deposition at enhancers. **A** Flowchart of experiments. **B** Number of differentially hydroxymethylated regions (DhmRs) comparing unstimulated vs. LPS-stimulated BMDMs. **C** LPS induces 5hmC deposition in BMDMs at an intergenic region between the *Jdp2* and *Batf* genes (for genome browser views of the *Il1b* and *Il6* loci, see Fig. S3). **D** For all the de novo 5hmC peaks in LPS-stimulated BMDMs, Log2 fold change in 5hmC is plotted against Log2 fold change in RNA expression. 5hmC peaks overlapping with latent enhancers (regions that acquire H3K4me1 and H3K27Ac only after stimulation) are shown in red; of these, peaks in the vicinity of the *Batf*, *Ptgs2*, *Stat1*, *Alcam*, *Mdfic*, *Il1b*, and *Il6* genes are shown in light blue. **E** Bar graphs show the RNA expression levels of the *Batf*, *Ptgs2*, *Stat1*, *Alcam*, *Mdfic*, *Il1b*, and *Il6* genes in *WT* vs. *TET iTKO* (*left*), or *WT* vs. *TDG iKO* (*right*), as determined by RNA-seq. BMDMs stimulated with (+) or without (−) LPS were used. **F** Heat maps depicting the percentage of (5mC+5hmC)/total Cs for each CpG in enhancers close to the *Batf*, *Ptgs2*, *Stat1*, *Alcam*, *Mdfic*, *Il1b*, and *Il6* genes, as determined by BS-seq. All seven selected latent enhancers show 5hmC deposition upon LPS stimulation (see Fig. S3E), but none undergoes DNA demethylation (loss of 5mC+5hmC)

Bone marrow-derived monocytes go through many cycles of cell division while they are differentiating into macrophages (bone marrow-derived macrophages (BMDM)) (Fig. 3A); however, differentiated BMDM do not proliferate further when stimulated with LPS [41]. We asked whether there was replication-independent (i.e., “active”) DNA demethylation under these conditions, and if so, whether it was mediated by TET and TDG. We began by mapping genome-wide changes in 5hmC by CMS-IP [42, 43]. Over a time course of 6 h of stimulation with LPS, differentiated BMDM acquired 4069 “de novo” 5hmC peaks compared to unstimulated cells (Fig. 3B, *blue wedge in the pie chart*). A subset (118) of these regions of “de novo” 5hmC deposition corresponded to so-called latent enhancers, which acquire the histone modifications H3K4me1 and H3K27Ac only after stimulation [22] (Fig. 3C, *red bar* in genome browser view of *Batf* locus; Fig. 3D, *red and light blue dots*). Motif enrichment analysis of either the 118 latent enhancers or all 4069 enhancers that acquired 5hmC progressively upon LPS stimulation showed that the most highly enriched motif was for consensus binding sites for NFκB (GGGAATTTCC or GGGAATTCCC; *data not shown*).

To investigate the roles of TET and TDG in DNA demethylation in these non-proliferating macrophages upon LPS stimulation, we selected the top five latent de novo enhancers that acquired 5hmC after LPS stimulation, located in or near the *Batf* (Fig. 3C), *Ptgs2*, *Stat1*, *Alcam*, and *Mdfic* (not shown) genes, as well as enhancers in the vicinity of the *Il1b* and *Il6* genes (Additional file 1: Fig. S3A, B) and determined their DNA modification before and after stimulation. *Batf* and *Ptgs2* were upregulated, whereas *Stat1*, *Alcam*, and *Mdfic* showed little or no change at the mRNA level in either WT or *TET iTKO* cells after LPS stimulation (Fig. 3D, *blue dots*; Fig. 3E). The genes encoding the cytokines *Il1b* and *Il6* were also substantially upregulated by LPS stimulation, more so in *TET iTKO* than in WT BMDM (Fig. 3D, E) and had latent enhancers in their vicinity (Additional file 1: Fig. S3A, B). Expression of the *Batf*, *Ptgs2*, *Il1b*, and *Il6* genes was increased in LPS-stimulated *TET iTKO* but not in LPS-stimulated *TDG iKO* BMDM compared to control LPS-treated BMDM (Fig. 3E; Additional file 1: S3C, D). However, none of these loci (i.e., the top five latent enhancers and the two close to the *Il1b* and *Il6* genes) showed changes in DNA methylation (5mC+5hmC) after 6 or 24 h of LPS stimulation, even though their basal methylation levels varied (Fig. 3F), indicating that the de novo gain of 5hmC at the latent enhancers observed in these gene loci is not linked to DNA demethylation. In *TDG iKO* cells, we also observed increased 5fC and 5caC after pyridine borane sequencing (Additional file 1: Fig. S3E). Inflammatory phenotypes (e.g., hyperproduction of IL-1β and IL-6) have previously been reported in *TET2*-mutated macrophages in humans [44]; however, the link between altered 5hmC deposition, TET dysfunction, and changes in gene expression still remains unclear. Our data indicate that TET proteins are recruited to enhancers that acquire 5hmC after LPS stimulation, most likely by NFκB, a known transcription factor that acts downstream of LPS [45]. Thus, at these locations, 5hmC represents an epigenetic mark whose function remains to be understood, rather than an intermediate in DNA demethylation.

### GATA3 is partly responsible for TET-dependent *Il4* gene demethylation

**Fig. 4:**
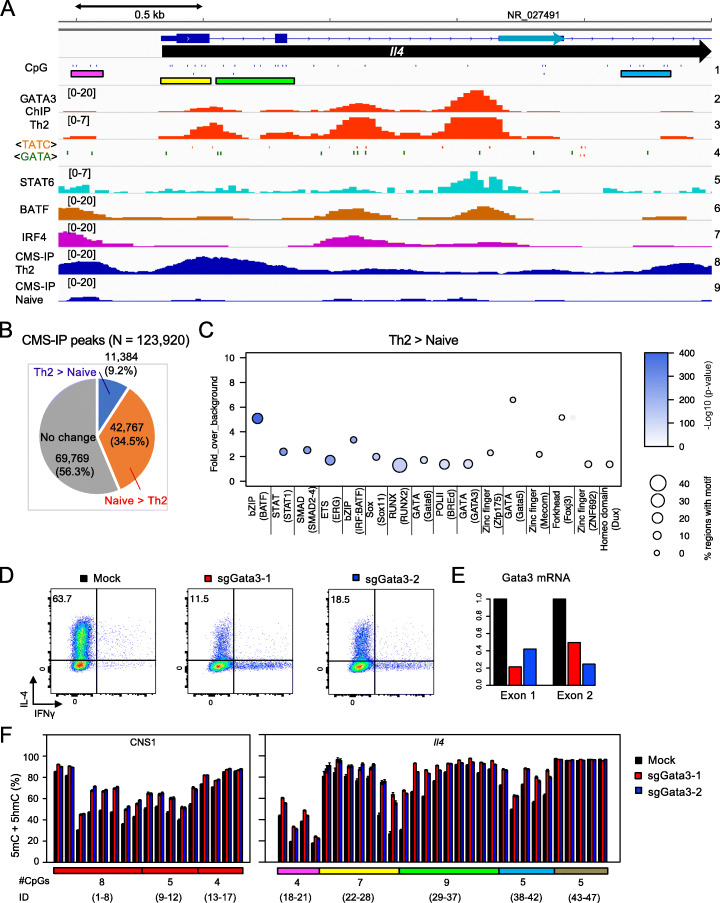
GATA3 is partly responsible for *Il4* locus demethylation. **A** Genome browser view of the 5′ half of the *Il4* gene. *Track 1*: CpGs are shown as short vertical blue lines. CpGs belonging to different amplicons are indicated by colored horizontal bars as in Fig. 1C [i.e., magenta bar for promoter CpGs, yellow bar for exon 1, green bar for intron 1-exon 2, etc.]. *Tracks 2 and 3*: GATA3 ChIP-seq profiles (GSE28292) at two different scales (0–20 and 0–7) in Th2 cells. *Track 4*: Positions of known GATA binding motifs (TATC and GATA). *Tracks 5–7*: ChIP-seq profiles for STAT6 (GSE22104), BATF (GSE85172), and IRF4 (GSE85172) in Th2 cells. *Tracks 8 and 9*: 5hmC profiles in Th2 and naïve CD4^+^ T cells respectively, determined by CMS-IP. Note the presence of 5hmC at regions occupied by transcription factors. The rightmost 5hmC/CMS-IP peak does not correspond to a region with GATA3, STAT6, BATF, or IRF4 occupancy. **B** Number of differentially hydroxymethylated regions (DhmRs) in naïve CD4^+^ T cells compared to Th2 cells. **C** Motif enrichment analysis of DhmRs that are enriched in Th2 cells compared to naïve T cells. The y axis indicates fold enrichment versus background, circle size indicates the percentage of regions containing the respective motif, and the color indicates the significance (-Log10 (p-value)). **D** Flow cytometry plots showing IL-4 and IFN-γ production by Th2 cells that were transduced with retroviral vectors encoding control or *Gata3* sgRNA. Data are representative of two independent experiments. **E** *Gata3* transcripts were quantified by qRT-PCR and normalized to *Gapdh* and then to the level of empty vector control. **F** A bar graph depicting the percentage of (5mC + 5hmC)/total C in 47 CpGs in the *Il4* locus in cells transduced/electroporated with control vs. *Gata3* sgRNA, as determined by BS-seq. Data are representative of two independent experiments

To identify transcription factors potentially responsible for TET-mediated demethylation, we performed 5hmC mapping in naïve CD4 T cells and differentiated Th2 cells by CMS-IP [42, 43] (Fig. 4A, *dark blue tracks 8 and 9*). Of a total of 123,920 CMS (5hmC)-enriched regions, 69,769 showed no difference in 5hmC in naïve versus Th2 cells, 42,767 regions lost 5hmC, and 11,384 regions gained 5hmC during Th2 differentiation (Fig. 4B; Additional file 1: Fig. S2B). Motif enrichment analysis of these 11,384 differentially hydroxymethylated regions (DhmRs) showed enrichment for consensus binding sequences for a variety of transcription factors including RUNX, STAT, SMAD, GATA, and basic region-leucine zipper (bZIP) transcription factors (Fig. 4C). Figure 4A (*tracks 2–7*) shows published ChIP-seq data for selected transcription factors known to be important in Th2 differentiation—GATA3, STAT6, the bZIP transcription factor BATF, and its partner IRF4. We focused on the Th2 lineage-determining transcription factor GATA3, which has a well-documented binding site in the first intron of the *Il4* gene (Fig. 4A, *red tracks 2 and 3*; *note different scales*) that overlaps with a large 5hmC peak identified by CMS-IP (Fig. 4A, *blue tracks 7 and 8*). Retroviral transduction of a vector containing *Gata3* sgRNA into naïve T cells isolated from *Cas9* transgenic mice led to decreased expression of GATA3 and consequently to decreased expression of IL-4 in the differentiated Th2 cells (Fig. 4D, E), compared to cells transduced with empty vector. Gata3-depleted cells also showed a substantial increase in DNA methylation (5mC+5hmC) in *CNS1* as well in the vicinity of the intronic Gata3 site, extending from the end of exon 1 (*yellow bar*) into intron 1 (*green bar*) of the *Il4* gene (CpGs 27–29) (Fig. 4F). Thus, like many other transcription factors, GATA3 (and potentially other unidentified transcription factors; see the “Discussion” section) promote DNA demethylation by recruiting TET enzymes to the regions they occupy in the *Il4* gene.

### The *Il4* gene is concordantly demethylated during Th2 cell differentiation

**Fig. 5:**
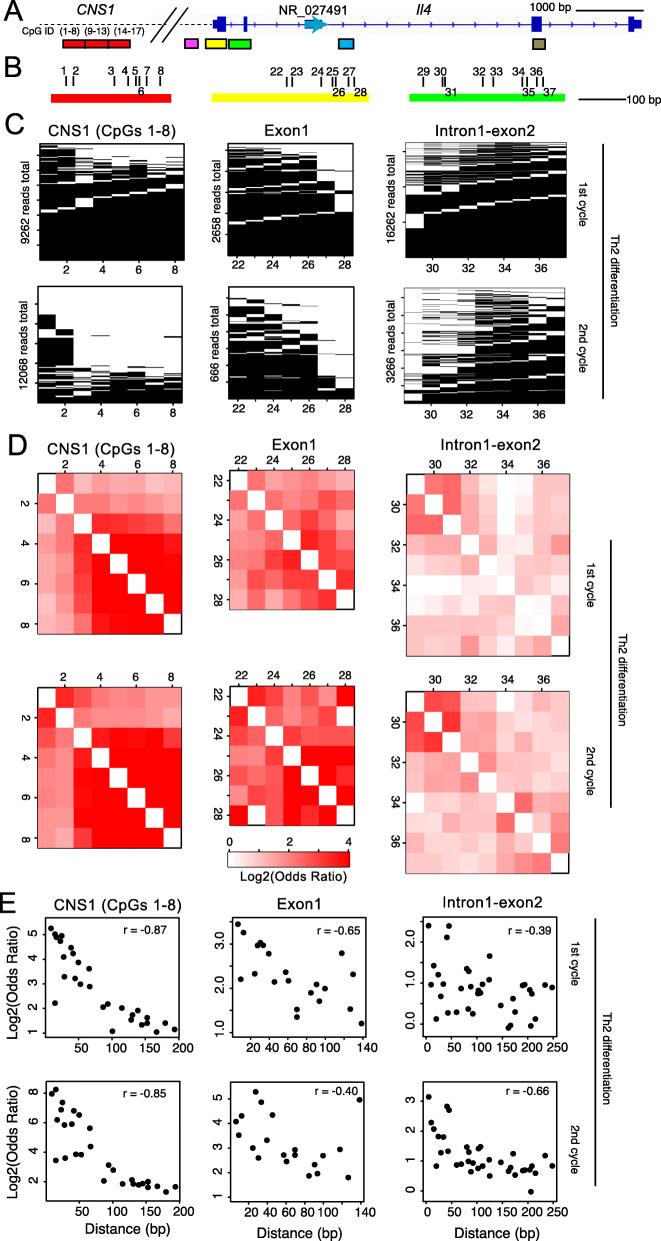
The *Il4* locus is concordantly demethylated during Th2 cell differentiation. **A** Genome browser view of part of the *Il4* locus, with amplicons indicated as in Fig. 1C. **B** Positions of CpGs in the first amplicon of *Il4 CNS1* (CpGs 1–8), and the amplicons for Exon 1 (CpGs 22–28) and Intron 1-Exon 2 (29–37). **C** Methylation profiles for *CNS1* (CpGs 1–8), Exon 1, and Intron 1-Exon 2 amplicons in Th2 cells after the first (*upper*) and second (*lower*) cycles of differentiation. Each row represents one read. Black indicates methylation (5mC + 5hmC) and white indicates the presence of unmodified C, 5fC, or 5caC at the indicated CpG. Note that demethylation occurs progressively between the first and second cycles of differentiation at all CpGs in the first amplicon of *Il4 CNS1* (CpGs 1–8), with the most extensive demethylation at CpGs 3–8; similarly, demethylation occurs progressively at the GATA3-binding intronic enhancer, with the most extensive demethylation occurring at CpGs 27–29 near the peak of GATA3 occupancy (see Fig. 4A). **D** Matrix showing odds ratio of any two CpGs as a measure of concordant modification in Th2 cells after the first (*upper*) and second (*lower*) cycles of differentiation. The brighter the red color, the more similar the methylation status of the CpGs being compared. The highest levels of concordant demethylation are observed at CpGs 3–8 of *Il4* CNS1, CpGs 25–28 of exon 1, and CpGs 29–31 of intron 1-exon 2. **E** For all possible pairs of CpGs, the odds ratio for a pair of CpGs is plotted against the distance between that pair of CpGs. Note that the odds Ratio (concordant methylation status) is higher the closer the CpGs

Since each cytosine in a CpG sequence in DNA is either modified or not, partial DNA methylation values actually represent averaged values of DNA modifications for all alleles in a population. Given that TET proteins are recruited to DNA by transcription factors, we asked whether demethylation was likely to be “concordant,” with closely spaced CpGs showing similar methylation/demethylation status [46]. To analyze such concordant methylation changes more precisely, we generated a methylation profile for each amplicon (Fig. 5A, B), in which each row corresponds to a single read (Fig. 5C). CpGs 3–8 in the *CNS1* enhancer and CpGs 25–31 in the exon 1/intron 1 enhancer which binds GATA3 [47] are fully methylated in naïve T cells (Fig. 2A) and become progressively demethylated with each cycle of Th2 differentiation (Fig. 5C).

To determine whether pairs of CpGs influenced each other, we developed a method to quantify the extent of concordant methylation in an amplicon. We prepared a 2 × 2 contingency table and counted the numbers of reads in which two CpGs in an amplicon were both methylated (MM), both unmethylated (UU), or only one was methylated (UM or MU) (Additional file 1: Fig. S4A, *upper-right panel*); in this table, the odds ratio, calculated as MM times UU over UM times MU, is equal to zero, one, or infinity when a given CpG pair is demethylated exclusively, independently, or concordantly, respectively, and can be used as a quantitative estimate of concordant demethylation. The results are shown in a matrix for each amplicon (Fig. 5D; Additional file 1: Fig. S4B-E); to illustrate, CpGs 27 and 28 exhibited odds ratios of 9.6 and 19.9 after one and two cycles of differentiation, respectively (Fig. 5D; Additional file 1: Fig. S4D). The concordant methylation status, judged by the odds ratio, is higher the closer the distance between any given pair of CpGs; this is particularly obvious for *CNS1* and the exon 1-intron 1 region (Fig. 5E; Additional file 1: Fig. S4E).

### TDG-deficient mice show normal hematopoiesis

**Fig. 6:**
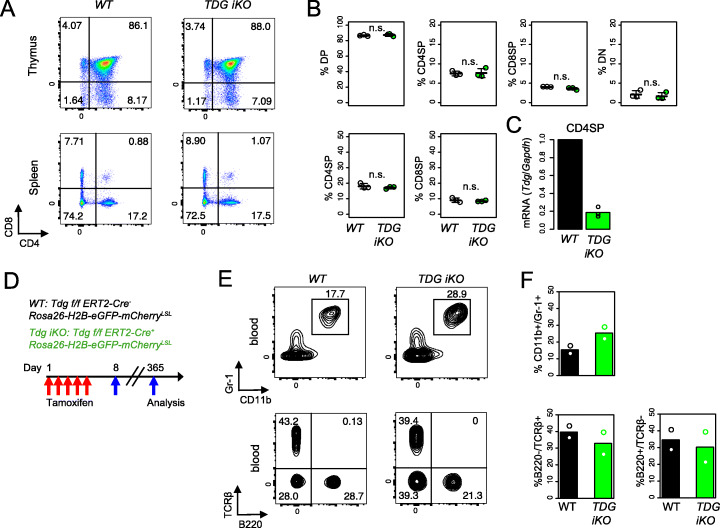
TDG-deficient mice show normal hematopoiesis. **A** Flow cytometry plots and **B** quantification of CD4 and CD8 in the thymus (*upper*) and spleen (*lower*) in *WT* (*n* = 3) and *TDG-KO* (*n* = 3) mice, in which *CD4Cre*-driven gene deletion was achieved. **C** CD4 single positive (SP) cells were isolated from the thymus. *Tdg* transcripts were quantified by qRT-PCR and normalized to *Gapdh* and then to the level of WT control. Data from three independent experiments with three technical replicates each. **D** Flowchart of experiments. **E** Flow cytometry plots and **F** quantification of CD11b, Gr-1, B220, and TCR β chain in cells in the peripheral blood of *WT* (*n* = 2) and *TDG iKO* (*n* = 2) mice. Statistical significance was calculated using an unpaired two-tailed t test

To evaluate the effect of *Tdg* deletion in T cell development, we generated *Tdg*^*fl/fl*^ *Cd4Cre Rosa26-H2B-EGFP/GPI-mCherry*^*LSL*^ mice in which loxP-flanked *Tdg* alleles (*Tdg*^*fl/fl*^) are deleted by Cre recombinase expressed from the T cell-specific *Cd4* promoter. Unlike *Tet2*^*fl/fl*^ *Tet3*^*fl/fl*^ *Cd4Cre* (*Tet2/3 Cd4Cre DKO*) mice, which display decreased percentages of DP thymocytes as well as a relative increase in the frequency of CD4SP and CD8SP cells in the thymus [48], TDG-deficient mice showed normal thymocyte development (Fig. 6A, B); the efficiency of *Tdg* deletion was high, with *Tdg* mRNA levels at ~20% of control (Fig. 6C).

To assess the biological role of TDG in hematopoietic development and function, we monitored *Tdg*^*fl/fl*^ *Cre-ERT2* mice after tamoxifen treatment. Previously, we had showed that adult mice with conditional inactivation of *Tdg* are viable up until 3 months post-tamoxifen treatment [49], but longer survival had not been evaluated. While *Tet2*^*fl/fl*^ *Tet3*^*fl/fl*^ *Cre-ERT2* mice developed progressive leukocytosis characterized by massive expansion of myeloid-lineage cells as early as 3–7 weeks after tamoxifen injection [50], *Tdg*^*fl/fl*^ *Cre-ERT2* mice were healthy and showed only a slight (not significant) increase in the myeloid population (CD11b^+^ Gr-1^+^) with a slight (not significant) decline in T and B cells in the peripheral blood at 1 year after tamoxifen injection (Fig. 6D–F). Together, these results demonstrate that loss of TDG in hematopoietic-lineage cells does not lead to major alterations in the bone marrow and thymus or in hematopoietic differentiation or function.

## Discussion

More than 10 years after the first report of TET enzymatic activity [9], it is clear that the vast majority of dynamic DNA demethylation in mammalian cells is mediated through the ability of TET enzymes to oxidize 5mC [3, 11]. However, for most biological systems of cell activation and differentiation, the extent to which DNA demethylation occurs through passive replication-dependent dilution of oxidized methylcytosines versus excision of 5fC and 5caC by TDG has been unknown.

In this study, we examined the relative roles of TET proteins and TDG, both in T cells that proliferate during the course of Th2 and iTreg differentiation, and in fully differentiated, bone marrow-derived macrophages that arrest proliferation upon activation with LPS. For T cell differentiation, we chose to focus on *Il4*, a key cytokine produced by differentiated Th2 cells [51]; we and others had already identified the major enhancers and silencers that controlled the expression of this cytokine gene [31, 52], and we had previously used much cruder techniques to show that the *Il4* gene underwent progressive demethylation during Th2 differentiation [21]. For LPS-activated macrophages, we focused on selected genes in the vicinity of “latent” enhancers [22] that acquired 5hmC as well as both H3K4me1 and H3K27Ac within 6 h after activation with LPS. In both systems, TDG indeed excises 5fC and 5caC, but this process makes only a minor contribution (if any) to the differentiation and activation of these cells (Figs. 1, 2, and 3). Moreover, *Tdg*^*fl*/fl^ *Cre-ERT2* mice that were rendered acutely TDG-deficient by treatment with tamoxifen in vivo survived normally for over a year, with only minor hematological abnormalities and no obvious propensity to disease (Fig. 6). These observations, together with other reports showing that adult mice subjected to acute *Tdg* deletion in vivo remain viable, healthy, and fertile [49, 53], suggest that TDG has very specific rather than widespread effects on DNA demethylation and gene expression in vivo.

TDG is the only DNA glycosylase whose germline deletion results in embryonic lethality [54–57]. However, *Tdg*-deficient mouse embryos survive until embryonic day 11.5, well past the earliest stages of dynamic DNA demethylation in the zygote and preimplantation blastocyst [58, 59]. In fact, zygotic DNA demethylation in both the paternal and maternal pronuclei was shown to occur largely in a passive replication-dependent manner, involving dilution of Tet3-generated oxi-mC as well as Tet3-independent dilution of 5mC [60, 61], the latter potentially explained by the fact that the oocyte-specific splice variant of the maintenance DNA methyltransferase Dnmt1 (termed Dnmt1o) is sequestered in the cytoplasm in oocytes and early embryos [62]. Even in mouse zygotes, however, some DNA demethylation occurred via an active, replication-independent process that was not blocked by aphidicolin [60, 61]. Curiously, however, this process of active demethylation—which required Tet3—did not appear to require TDG, suggesting the participation of other biochemical pathways for effecting DNA demethylation by removing oxi-mCs.

Nevertheless, based on the increase in 5fC and 5caC and DNA methylation (5mC+5hmC) in numerous *Tdg*-deficient cell types in mice—embryonic stem cells [40, 63–66], embryos and embryonic fibroblasts [53, 55, 56], maternal and paternal pronuclei in the zygote [6, 60, 67], brain [68], and immune cells [this study]—TDG clearly does excise these oxi-mC bases in vivo. Only in a few cases—for instance, the retinoic acid-induced upregulation of the *Hic1* gene in mouse embryonic fibroblasts [53] and upregulation of the genes encoding the miR-200 microRNA family during somatic cell reprogramming with the 3 Yamanaka factors *Oct4*, *Sox2*, and *Klf4* [39]—were both gene expression and DNA methylation status shown to be sensitive to the loss of TDG. Thus, despite the demonstration of TET-TDG interaction and the elegant biochemical reconstitution of DNA demethylation in vitro by TET, TDG, and enzymes required for base excision repair [69], TDG does not appear to be required in vivo either for the intensively studied process of zygotic DNA demethylation or for the somatic cell activation and differentiation in T cells and macrophages that we have examined here.

In contrast, numerous studies have shown that DNA demethylation and gene expression are linked to TET activity and that TET proteins are recruited by transcription factors to promoters, enhancers, and other regions that undergo demethylation [23, 35, 70–72]. In line with this conclusion, we show here that CRISPR-mediated depletion of GATA3, a transcription factor essential for Th2 differentiation, results in increased DNA methylation around a well-established GATA3 binding site in the first intron of the *Il4* gene (Fig. 4). However, we did not observe co-immunoprecipitation of GATA3 and TET2 (*not shown*). In a breast cancer cell line, TET2 was reported as a component of the estrogen receptor (ER)/GATA3 complex and was shown to be recruited in a GATA3-dependent manner [73]. Direct co-immunoprecipitation of GATA3 and TET2 was not tested in this study; however, TET2 was recruited to target sites by forced expression of a mutant GATA3, indicating that GATA3 may contribute indirectly to recruitment of TET2 to DNA.

We also identified a region (HSII 3′ region) in the second intron of the *Il4* gene that became demethylated during Th2 differentiation, in a manner unaffected by the loss of either TET or TDG (Fig. 1). The mechanism of TET-independent demethylation of this HSII region is not yet understood. It seems unlikely that the estimated ~2-fold decrease in DNMT1 expression in *TET iTKO* compared to WT Th2 cells accounts for specific TET-independent demethylation of only the 5 CpGs present in this region. The HSII 3′ region overlapped partially with a region enriched for 5hmC in Th2 cells (Fig. 4A), suggesting that it might be bound by a different transcription factor that recruited TET proteins. There was no apparent binding of GATA3 to this region (Fig. 4A), but its methylation level was somewhat increased by GATA3 depletion (Fig. 4D–F). The region also lies 3′ of a non-coding RNA, NR_027491, located in the *Il4* second intron (shown as a blue bar within intron 2 of the *Il4* gene in Fig. 4A). Thus, DNMT1 may be excluded from the DNase HSII 3′ region either because the region is bound by the unidentified transcription factor, or alternatively, because of the presence of the adjacent annotated non-coding RNA, NR_027491. Indeed, there are many reports showing that non-coding transcripts can interact with chromatin modifiers and promote or inhibit their recruitment to target DNA [74]. Particularly, DNMT1 can be inhibited by non-coding RNAs via direct binding in several cell lines [75–77]. In addition, genetic deletion of the *Dnmt1* gene results in decreased methylation levels at the *Il4* promoter and the *Il4 CNS1* region even in CD8^+^ T cells [78].

Conventional whole-genome bisulfite sequencing can provide information on the proportion of methylated cytosine (5mC + 5hmC) at each CpG that is covered by a sufficient number of reads (5–15x coverage) [79]; however, it is not straightforward to analyze how DNA methylation at any given CpG relates to methylation at adjacent CpGs. This point is important to understand because even with the same methylation rate (e.g., 50%), whether demethylation occurs exclusively, randomly, or concordantly (Additional file 1: Fig. S4A, *left panels*) could affect the biological output. For example, some regions display different DNA methylation patterns between normal and cancer tissues in the context of concordance, but the same average methylation level [80]. In this study, we successfully quantified the degree of concordant demethylation by using odds ratio with short PCR amplicon-based analysis of the *Il4* gene. We found that the closer the distance, the more concordant demethylation occurred and that there were “blocks” within which concordant demethylation was observed (Fig. 5D). Our analysis provides direct evidence that changes in epigenetic modification are actually occurring concordantly. This odds ratio-based analysis would also be useful for analyzing data from long-read DNA methylation sequencing [81].

DNA methylation levels at promoters show a clear inverse correlation with gene expression, but there has been some controversy about whether promoter demethylation causes or follows increased gene expression. The arguments for DNA demethylation being driven by transcription factors and gene transcription have been persuasively summarized [29, 30]. On the other hand, many studies—including those that have targeted TET proteins to specific genomic regions using inactive Cas9 proteins—have concluded that DNA demethylation can activate gene expression. Our explanation for this paradox is that TET-dependent demethylation enhances target expression and provides a positive feedback loop in which initial binding of transcription factors to an enhancer region promotes recruitment of TET proteins as well as chromatin remodeling complexes, which in turn increase the accessibility of the region for binding of more and/or additional transcription factors, which promotes more TET recruitment and more DNA demethylation. To illustrate, in *TET iTKO* Th2 cells, we observed a marked decrease in IL-4 expression; however, these cells were still capable of producing significant amounts of IL-4. This result suggests that TET proteins are not necessary for the initial activation of the *Il4* gene but rather promote optimal expression of IL-4. A likely mechanism would be as follows: first, GATA3 remodels and activates the *Il4* gene; second, demethylation is induced as a consequence in a TET-dependent manner in most regions except the HSII 3′ region; third, demethylation enhances IL-4 expression and Th2 cells achieve optimal IL-4 expression.

## Conclusions

Based on these data, we conclude that TDG has a minor role in active replication-independent DNA demethylation, both during Th2 differentiation and macrophage activation. DNA demethylation during Th2 differentiation occurs mainly through passive replication-dependent dilution of all three oxidized methylcytosines, with only a negligible contribution from TDG. In an independent system that does not involve cell division, macrophages treated with LPS for 6 h accumulate 5hmC at enhancers and show altered gene expression without DNA demethylation; loss of TET enzymes disrupts gene expression, but loss of TDG has no effect. Our study complements and corroborates previous studies in other biological systems, showing that DNA methylation in somatic cells is almost invariably removed through a TET-dependent process of “passive” DNA demethylation, in which active excision of 5fC and 5caC by TDG has only a marginal role.

## Methods

### Mice

*Tet1*^fl/fl^, *Tet2*^fl/fl^, and *Tet3*^fl/fl^ (Tet triple floxed) mice were generated as previously described [82]. *Tdg*^fl/fl^ mice were generated as previously described [83]. C57BL/6J (000664), *Ubc-CreERT2* (008085; located at Ndor1 locus; described as *CreERT2* herein), *Rosa26-EYFP*^*LSL*^ (006148), *Rosa26-H2B-EGFP/GPI-mCherry*^*LSL*^ (021847), and *Rosa26-Cas9* (028555) were obtained from the Jackson Laboratory. The LSL (LoxP-STOP-LoxP) cassette in *Rosa26-EYFP*^*LSL*^ or *Rosa26-H2B-EGFP/GPI-mCherry*^*LSL*^ mice contains a strong transcriptional stop flanked by two LoxP sites and was used as an indicator of the Cre activity. All mice used were 8 to 16 weeks in the C57BL/6 background and kept in a specific pathogen-free animal facility at La Jolla Institute and were used according to protocols approved by the Institutional Animal Care and Use Committee. To induce *CreERT2*-mediated deletion, we intraperitoneally injected Cre-expressing and control mice with 2 mg of tamoxifen (Sigma) dissolved in 100 μl of corn oil (Sigma). The extent of deletion of the floxed exons of *Tet1*, *Tet2*, *Tet3*, and *Tdg*, measured by qRT-PCR after 1 cycle of Th2 differentiation, was 96%, 80%, 96%, and 92%, respectively.

### Flow cytometry and FACS

The antibodies used for naive CD4^+^ T cell staining from the spleen and lymph nodes were BV421-conjugated anti-CD4 mAb (GK1.5; BioLegend), APC-conjugated anti-CD62L mAb (MEL-14; BioLegend), PE-conjugated anti-CD44 mAb (IM7; BioLegend), and PECy5-conjugated anti-CD25 mAb (PC61; eBioscience). The antibodies used for intracellular staining were BV421 or AF700-conjugated anti-CD4 mAb (GK1.5; BioLegend), APC-conjugated anti-IL-4 (11B11; eBioscience), PE-conjugated anti-IFN-γ (XMG1.2; BioLegend), AF647-conjugated anti-GATA3 (L50-823; BD Pharmingen), and PE-conjugated anti-Foxp3 (FJK-16s; eBioscience). The antibodies used for surface staining of thymocytes and splenocytes were BV421-conjugated anti-CD4 mAb and AF700-conjugated anti-CD8a mAb (53-6.7; BioLegend). The antibodies used for surface staining of the peripheral blood were Pacific Blue-conjugated anti-Gr-1 mAb (RB6-8C5; BioLegend), PerCP/Cyanine5.5-conjugated anti-CD11b mAb (M1/70; BioLegend), APC-conjugated anti-B220 mAb (RA3-6B2; BioLegend), and PE-conjugated anti-TCR β chain mAb (H57-597; BioLegend). For cell surface staining, cells were stained in fluorescence-activated cell sorting (FACS) buffer (1% bovine serum albumin, 1 mM EDTA, and 0.05% sodium azide in PBS) with indicated antibodies for 30 min on ice. Cells were washed and then underwent flow cytometric analysis using FACS LSR II instrument (BD Biosciences), and the results were analyzed with the FlowJo software program (BD Biosciences).

### In vitro Th2 cell differentiation (1st and 2nd cycle), iTreg differentiation

Total CD4^+^ T cells were isolated with the EasySep Mouse CD4^+^ T cell isolation kit (STEMCELL Technologies, Canada) from the spleen and lymph nodes. Then, CD4^+^CD25^−^CD62L^hi^CD44^lo^ (EYFP^+^ or EGFP^+^ for *Rosa26-EYFP*^*LSL*^ and *Rosa26-H2B-EGFP/GPI-mCherry*^*LSL*^ mice, respectively) naive CD4^+^ T cells were FACS sorted. For Th2 polarization, naïve CD4^+^ T cells were stimulated with plate-bound anti-CD3 (clone 2C11) and anti-CD28 (clone 37.51) antibodies at 1 μg/ml in the presence of 1000 U/ml IL-4 and 1 μg/ml anti-IFN-γ, for 2 days. On day 3 of culture, cells were removed from TCR/CD28 costimulation conditions and expanded in the presence of 20 U/ml IL-2 for 4 days. For the 2nd cycle of the cultivation [84], Th2 cells differentiated for 6 days were washed and cultured under resting conditions (medium without any cytokine) for 1 day. Cells were harvested, counted, and plated at 10^6^ cells/ml for 2nd stimulation with plate-bound anti-CD3 (clone 2C11) and anti-CD28 (clone 37.51) antibodies at 1 μg/ml in the presence of 1000 U/ml IL-4 and 1 μg/ml anti-IFN-γ, for 1 day. On day 2 of the 2nd cycle, cells were removed from TCR/CD28 costimulation conditions and expanded in the presence of 20 U/ml IL-2 for 4 days. For iTreg differentiation, naïve CD4^+^ T cells were stimulated with plate-bound anti-CD3 (clone 2C11) and anti-CD28 (clone 37.51) antibodies at 1 μg/ml in the presence of 10 ng/ml recombinant human TGF-β (PeproTech) and 100 U/ml recombinant human IL-2 (rhIL-2). On day 3 of culture, cells were removed from TCR/CD28 costimulation conditions and expanded in the presence of 100 U/ml IL-2 for 2 days.

### Th2 cell differentiation and restimulation for assessing GATA3 protein stability

Differentiated Th2 cells were harvested, counted, and plated at 10^6^ cells/ml for 2nd stimulation with plate-bound anti-CD3 and anti-CD28 antibodies at 1 μg/ml in the presence of 1000 U/ml IL-4, 1 μg/ml anti-IFN-γ, and 2 μM 4-hydroxytamoxifen (4-HT) for 1 day. One day after restimulation, cells were removed from TCR/CD28 costimulation conditions and expanded in the presence of 20 U/ml IL-2 for 4 days. Five days after restimulation, GATA3 expression was examined by intracellular staining.

### In vitro iTreg cell differentiation and restimulation for assessing Foxp3 protein stability

Naive T cells were differentiated into iTreg cells with plate-bound anti-CD3 and anti-CD28 antibodies at 1 μg/ml in the presence of 2 ng/ml recombinant human TGF-β, 100 U/ml recombinant human IL-2, 100 nM RA (Sigma-Aldrich), and 100 μg/ml vitamin C (Sigma-Aldrich) [24]. For the restimulation experiments, iTreg cells differentiated for 6 days were harvested, counted, and plated at 0.8 × 10^6^ cells/ml for restimulation with plate-bound anti-CD3 at 50 ng/ml and anti-CD28 at 25 ng/ml in the presence of 2 μM 4-HT. One day after restimulation, cells were removed from TCR/CD28 costimulation conditions and expanded in the presence of 100 U/ml IL-2 for 2 days. Three days after restimulation, Foxp3 expression was examined by intracellular staining.

### Intracellular staining for IFN-γ and IL-4

On day 4 or 5 of in vitro culture, cells were restimulated for 4 h in the presence of 10 nM PMA (Calbiochem), 500 nM ionomycin (Calbiochem), and 2 μM Monensin. Harvested cells were fixed with 4% (wt/vol) paraformaldehyde (PFA) for 10 min at room temperature and permeabilized in a permeabilizing solution (50 mM NaCl, 5 mM EDTA, 0.02% NaN3, pH 7.5) containing 0.5% Triton X for 10 min on ice. After blocking with FACS buffer for 15 min, cells were incubated on ice for 30 min with appropriate staining antibodies. Cells were washed with FACS buffer at the end of each step. Flow cytometry was performed on a FACS LSR II instrument (BD Biosciences), and the results were analyzed with the FlowJo software program (BD Biosciences).

### Bone marrow-derived macrophage (BMDM) culture

The BMDMs were generated as described previously [85]. Femur, tibia, and iliac bones from the different mouse strains were flushed with DMEM, high glucose (GIBCO), and red blood cells were lysed using red blood cell lysis buffer (eBioscience). After counting, 10 million bone marrow cells were seeded per 10-cm non-tissue culture plates in DMEM high glucose (50%) with 20% FBS, 30% L929-cell-conditioned laboratory-made media (as source of M-CSF), and 100 U/ml penicillin/streptomycin+L-glutamine (GIBCO). After 4 days of differentiation, 20 ng/ml mouse M-CSF (Shenandoah Biotechnology) was added to the media. After an additional 2 days of culture, non-adherent cells were washed off with room temperature DMEM and macrophages were obtained as a homogeneous population of adherent cells which were scraped and subsequently seeded onto tissue culture-treated Petri dishes overnight in DMEM containing 20% FBS, 30% L929-cell-conditioned laboratory-made media, and 100 U/ml penicillin/streptomycin+L-glutamine. For KLA activation, macrophages were treated with 10 ng/ml KLA (Avanti Polar Lipids) for 6 h.

### RNA extraction, cDNA synthesis, and qRT-PCR

Total RNA was isolated with RNeasy Plus Kit (Qiagen, Germany) or with Trizol (Thermo Fisher Scientific, Waltham, MA, USA) following the manufacturers’ instructions. cDNA was synthesized using SuperScript II reverse transcriptase (Thermo Fisher Scientific), and qRT-PCR was performed using SYBR® Select Master Mix (Thermo Fisher Scientific) on a StepOnePlus Real-time PCR system (Thermo Fisher Scientific). Gene expression was normalized to Gapdh. Primers are listed in Table S1.

### Bisulfite sequencing (BS-seq)

Genomic DNA (gDNA) was isolated by PureLink Genomic DNA Mini Kit (Thermo Fisher Scientific). 0.5 μg of gDNA samples was treated with sodium BS (MethylCode Bisulfite Conversion kit; Invitrogen). We designed 5 primer pairs, which cover 30 CpGs (#18-47) within and around the *Il4* gene, as well as 6 primer pairs, which cover 34 CpGs in two conserved non-coding sequences (CNS), CNS1/HSS 3 (#1-17) and CNS2/site V (#48-64), and analyzed methylation status in naïve CD4 T cells as well as Th2 cells from WT, *TET iTKO*, and *TDG iKO* cells after 1 and 2 cycles of differentiation. We also designed 7 primer pairs, which cover 35 CpGs within the 7 latent enhancers in BMDMs. PCR primers (Table S1) were designed using MethPrimer (http://www.urogene.org/methprimer). The PCR amplicons were generated using the PyroMark PCR kit (QIAGEN) and quantified using Qubit assays (Invitrogen). PCR amplicons were pooled and then used for library preparation using NEB Next DNA Library Modules for Illumina platform (New England Biolabs, Inc.). The final libraries were quantified using the KAPA library quantification kit for Illumina (KAPA Biosystems) and sequenced on Miseq (250 bp, paired end; Illumina) or NovaSeq 6000 (150 bp, paired end; Illumina). The data are based on more than hundreds of sequence reads per amplicon, and a rigorous statistical analysis of 5mC/5hmC (detected as C) and C/5fC/5caC (detected as T) levels was performed as described in the section following the next one. BS conversion efficiency assessed by non-CpG sites is shown in Table S2.

### Pyridine borane sequencing (PB-seq)

One hundred nanograms of gDNA in 35 μl of water were reduced in a 50-μl reaction containing 600 mM sodium acetate solution (pH = 4.3) and 1 M pyridine borane (SIGMA or Alfa Aesar) for 16 h at 37°C and 850 rpm in Eppendorf ThermoMixer [37]. The product was purified by PureLink Genomic DNA Mini Kit (Thermo Fisher Scientific). PCR, library preparation, and sequencing were performed as described in the section above. The data are based on more than hundreds of sequence reads per amplicon, and a rigorous statistical analysis of C/5mC/5hmC (detected as C) and 5fC/5caC (detected as T) levels was performed as described in the following section. PB conversion efficiency assessed by spike-in control is shown in Table S3.

### BS-seq and PB-seq data analysis

The BS and PB reads were mapped to mouse genome mm9 using the BSMAP mapping tool [86]. The mapping was done using the paired end datasets with the following parameter values: -p 4 -w 2 -v 5 -q 30 for BS-seq, and -p 4 -w 2 -v 0 -q 30 for PB-seq. For each of the samples, the number of reads in which each cytosine within the amplicons was converted into thymine was counted. For BS-seq, these counts were used to calculate the proportions for each cytosine to be nonmethylated/formylated/carboxylated (T) or methylated/hydroxymethylated (C). For PB-seq, to compensate redundant reads derived from the opposite strand, the number of reads standing for nonmethylated/methylated/hydroxymethylated (C) was multiplied by 0.5, and the proportions for each cytosine to be formylated/carboxylated (T) was calculated. The confidence interval of the proportions was determined based on the model of a binomial distribution. For the concordant analysis, numbers of reads in which two CpGs in an amplicon are both methylated (MM), both unmethylated (UU), or one unmethylated but the other methylated (UM or MU) were counted. The odds ratio was calculated as MM times UU over UM times MU.

### Genome-wide 5hmC mapping by CMS-IP

CMS-IP was performed essentially as previously described [42]. Briefly, genomic DNA isolated from CD4^+^ T cells or BMDMs was spiked with unmethylated lambda phage cI857 Sam7 DNA (Promega, Madison, WI, USA) at a ratio of 200:1. DNA (5 to 10 μg in 130 μl tris-EDTA buffer) was sheared with a Covaris E220 using microTUBE for 4 min. DNA was cleaned up with Ampure XP beads, processed with NEBNext End Repair and A-tail Modules (NEB, Ipswich, MA, USA), and ligated to methylated Illumina adaptors (NEB). DNA was then bisulfite-treated (MethylCode, Thermo Fisher Scientific), denatured, and immunoprecipitated with anti-CMS serum (in-house) and a mixture of protein A and G Dynabeads (Thermo Fisher Scientific). Libraries for immunoprecipitated DNA were generated by PCR with barcoded primers (NEBNext Multiplex Oligos for Illumina; NEB) for 15 cycles using KAPA HiFi HotStart Uracil+ ReadyMix (Roche), followed by a cleanup with Ampure XP beads (Beckman Coulter), and sequenced with a HiSeq 2500 (Illumina, San Diego, CA, USA) with paired-end 50-bp reads. Reads were mapped to mouse genome assembly version of July 2007 (NCBI37/mm9) using the BSMAP mapping tool. The mapping was done using the paired end datasets with the following parameter values: -p 8 -w 100 -v 5. Enriched regions relative to input DNA were detected using the “findPeaks” routine in HOMER [87] with the “histone” mode and default parameter values. Differentially hydroxymethylated regions (DhmRs) were detected using the “getDifferentialPeaks” routine in HOMER.

### RNA-seq

Total RNA was isolated with a combination of RNeasy Plus Kit (Qiagen, Germany) and Trizol (Thermo Fisher Scientific, Waltham, MA, USA) following the manufacturers’ instructions. The integrity of the RNA was accessed with TapeStation RNA analysis ScreenTape (Agilent). Libraries were prepared using the NEBNext® Ultra II Directional RNA Library Prep Kit for Illumina (NEB). The starting RNA material was 200 ng. Briefly, polyA+ RNAs were selected with magnetic beads, the RNA was fragmented and cDNA was synthesized. After A-tailing and adaptor ligation, libraries were generated by amplifying the cDNA for 10–12 cycles and purified with Ampure XP beads. Libraries were sequenced on an Illumina HiSeq 2500 or NovaSeq 6000 with pair-end 50-bp reads. RNA-seq data were aligned to the mouse reference genome (mm9) by Hisat2 [88]. Gene expression was summarized by HTSeq-counts [89]. The DESeq2 package v1.30.0 [90] was used to normalize the raw counts and identify differentially expressed genes (adjusted P-value < 0.05).

### Retroviral transduction for sgRNA-mediated gene deletion

MSCV-pU6-(BbsI)-CcdB-(BbsI)-Pgk-Puro-T2A-BFP was purchased from Addgene (86457). sgRNA for the *Gata3* gene was designed as previously described [91] and cloned into the plasmid. Retrovirus was produced by transfecting PlatE cells with murine stem cell virus–based retroviral vectors and pCL-Eco. Naïve CD4^+^ T cells from Rosa26-Cas9 mice were stimulated with plate-bound anti-CD3 (clone 2C11) and anti-CD28 (clone 37.51) antibodies at 1 μg/ml in the presence of 1000 U/ml IL-4, 1 μg/ml anti-IFN-γ, at 1 × 10^6^ cells/ml for 24 h. Retrovirus was added to the cells in the presence of 20 mM Hepes and Polybrene (0.8 μg/ml; Millipore) and centrifuged at 2000 rpm at 32°C for 90 min. Cells were stimulated with plate-bound anti-CD3 and anti-CD28 antibodies at 1 μg/ml in the presence of 1000 U/ml IL-4 for another 24 h. On day 3 of culture, cells were removed from TCR/CD28 costimulation conditions and expanded in the presence of 20 U/ml IL-2 for 3 days. On day 6 of the culture, CD4^+^GFP^+^EBFP^+^ cells were sorted for qRT-PCR or BS-seq.

### Immunoblotting

Proteins isolated from T cells or BMDMs with radioimmunoprecipitation assay (RIPA) buffer and 140 U/ml Benzonase (Merck Millipore) were resolved using NuPAGE 4 to 12% bis-tris gel (Thermo Fisher Scientific) and transferred from gel to polyvinylidene difluoride membrane using Wet/Tank Blotting Systems (Bio-Rad). Membrane was blocked with 5% nonfat milk (Bob’s Red Mill) in TBS-T buffer [50 mM tris-HCl (pH 7.4), 150 mM NaCl, and 0.05% Tween 20] and incubated with indicated primary antibodies, followed by secondary antibodies conjugated with horseradish peroxidase (HRP), and the signal was detected with enhanced chemiluminescence reagents and x-ray film. The antibodies used for immunoblotting were anti-Dnmt1 Ab (ab19905; Abcam), HRP-conjugated anti-β-Actin Ab (#5125S; Cell Signaling), anti-Uhrf1 mAb (D6G8E; Cell Signaling), and HRP-conjugated anti-Rabbit IgG Ab (#7074; Cell Signaling).

### Statistical analyses

Statistical analyses and bar plots were performed and plotted with R (v3.6.1). Most experiments were analyzed using a two-tailed unpaired t-test, as indicated in the figure legends unless otherwise stated. The confidence interval of the proportions was determined based on the model of a binomial distribution.

## Supplementary Information

**Additional file 1: Supplementary Figures S1-S4.**

**Additional file 2: Table S1.** Primer sequences used for various PCR-based analyses.

**Additional file 3: Table S2.** BS conversion efficiency assessed by non-CpG sites.

**Additional file 4: Table S3.** Pyridine Borane conversion efficiency assessed by spike-in control.

**Additional file 5.** Review history.

## Data Availability

All genome-wide sequencing datasets have been deposited to Gene Expression Omnibus (GEO) repository, accession number GSE59213 [92] and GSE163895 [93].
