# Supplementary material for: Roles of TET and TDG in DNA demethylation in proliferating and non-proliferating immune cells
Source: Genome Biol. 2021 Jun 22;22:186. doi: 10.1186/s13059-021-02384-1 (PMC8218415; doi:10.1186/s13059-021-02384-1)
Supplement: Supplementary file 1 — Additional file 1: Supplementary Figures S1-S4. [file 13059_2021_2384_MOESM1_ESM.docx]

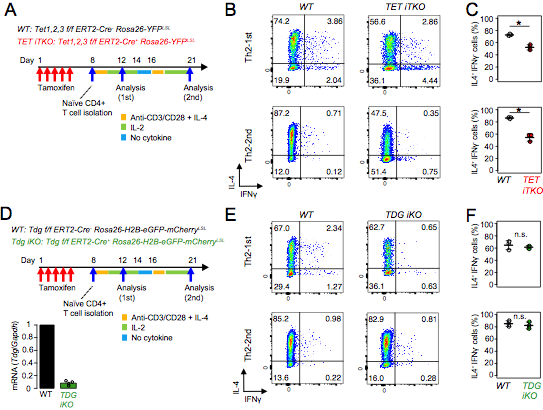

**Figure S1.** **TET enzymes are important, but TDG is dispensable, for IL-4 production by Th2 cells and** **Foxp3 expression by iTreg cells**

(**A**) Flowchart of experiments with *WT* and *TET iTKO* Th2 cells. (**B**) Flow cytometry plots and (**C**) quantification of IL-4 production by Th2 cells from *WT* (n = 3) and *TET iTKO* (n = 3) mice. TET deficiency results in decreased IL-4 production after both the first and second cycles (adapted from Fig. 1B) of Th2 differentiation. (**D**) Flowchart of experiments with *WT* and *TDG iKO* Th2 cells. (**E**) Flow cytometry plots and (**F**) quantification of IL-4 production by Th2 cells from *WT* (n = 3) and *TDG iKO* (n = 3) mice. TDG deficiency has no effect on IL-4 production after either the first or second cycle (adapted from Fig. 1B) of Th2 differentiation. (**G**) Flowchart of experiments with *WT* vs. *TDG iKO* or *WT* vs. *TET iTKO* Th2 cells, performed after two cycles of differentiation. 4-hydroxytamoxifen (4-HT) was added during restimulation. (**H**) Flow cytometry plots (upper) and quantification of GATA3 mean fluorescence intensity (MFI) (lower) in *WT* (n = 2) vs. *TDG iKO* (n = 2) or *WT* (n = 2) vs. *TET iTKO* (n = 2). (**I**) Flowchart of experiments with *WT* and *TDG iKO* iTreg cells, performed after one cycle of differentiation. (**J**) Flow cytometry plots and (**K**) quantification of Foxp3-expressing iTreg cells induced by IL-2 and the indicated concentrations of TGF-β in *WT* (n = 3) and *TDG iKO* (n = 3) mice. TDG deficiency has no effect on Foxp3 expression by iTreg cells. (**L**) Flowchart of experiments with *WT* and *TDG iKO* iTreg cells, performed three days after restimulation. (**M**) Flow cytometry plots (left) and quantification of Foxp3 MFI (right) in *WT* (n = 2) vs. *TDG iKO* (n = 2) mice. (**N**) *Left*, Analysis of cell proliferation by CTV dilution in differentiating naïve T cells from *WT* (*black*) vs. *TET iTKO* (*red*) Th2 cells. *Right*, Percentages of IL-4-producing cells in each population (divisions 1 to 5) are shown. Data are representative of two independent experiments. (**O**) Bar graphs show the percentage of (5mC + 5hmC)/total C in 17 CpGs in the *Il4* *CNS2* (HSV) region with confidence intervals (CIs) in *WT* vs. *TET iTKO* (*left*), or *WT* vs. *TDG iKO* (*right*), as determined by BS-seq. Results for naïve CD4^+^ T cells and Th2 cells after two differentiation cycles are shown. Data are representative of two independent experiments. TET deficiency impairs DNA demethylation of CpGs at the edges of *CNS2*, whereas TDG deficiency has no effect. (**P**) Flowchart of experiments with *WT* vs. *TDG iKO* or *WT* vs. *TET iTKO* Th2 cells, performed after one cycle of differentiation. (**Q**) Immunoblotting of whole-cell lysates with indicated antibodies. β-Actin was used as loading control. Statistical significance was calculated using unpaired two-tailed t test. **p* < 0.05.

**Figure S2. TDG deficiency does not affect gene expression in Th2 cells, and genomic distribution of** **5hmC in WT Th2 cells (related to Figures 1 and 2)**

(**A**) MA-plot depicts gene expression levels in *WT* vs. *TDG iKO* Th2 cells, as determined by RNA-seq. There is no change in gene expression in *TDG iKO* cells compared to *WT*. (**B**) *Left* and *middle*, Pie charts showing the overall enrichment of 5hmC-containing regions in different genomic contexts – transposable elements (LINEs, SINEs and LTRs), intergenic regions and gene-associated regions (promoters, exons and introns) – in Th2 cells compared to their representation in the mm9 genome (log2 ratios of observed over their representation in the genome).

**Figure S3.** **LPS stimulation of BMDMs induces 5hmC deposition at the *Il1b* and *Il6* enhancers (related to Figure 3)**

(**A**, **B**) LPS induces 5hmC deposition in BMDMs at intergenic latent enhancers in the vicinity of the *Il1b* (**A**) or *Il6* (**B**) genes. For the *Il1b* gene, the right-most latent enhancer was selected for further analysis. (**C**) MA-plot depicts gene expression levels in *WT* vs. *TET iTKO* (*left*) or *WT* vs. *TDG iKO* (*right*) BMDMs after stimulation with LPS for 6 h. (**D**) *Batf*, *Il1b*, *Il6*, *Tet1*, *Tet2*, *Tet3*, and *Tdg* transcripts were quantified by qRT-PCR and normalized to *Gapdh* and then to the level of WT control. Representative data of two independent experiments with three technical replicates. (**E**) Bar graphs show the percentages of (5fC + 5caC)/total C in 7 selected CpGs that can be analyzed by 50 bp pair-end reads in enhancers close to the *Batf*, *Mdfic*, *Il1b*, and *Il6* genes, in *WT* vs. *TDG iKO* BMDMs, as determined by PB-seq. Data are representative of two independent experiments.

**Figure S4.** **The *Il4* locus is concordantly demethylated during Th2 cell differentiation (related to Figure 5)**

(**A**) *Left*, Calculation of concordant demethylation for a given CpG pair (X and Y). Filled and empty circles represent methylated and unmethylated CpGs, respectively. Rows represent methylation patterns of each sequencing read. Three extreme examples are shown: fully exclusive (*left*), random (*middle*), and fully concordant (*right*). *Upper* *right*, A 2x2 contingency table showing numbers of reads in which two CpGs in an amplicon are both methylated (MM), both unmethylated (UU), or one unmethylated but the other methylated (UM or MU). In this table, the Odds ratio (OR) is calculated as MM times UU over UM times MU. The Odds ratio is equal to zero, one, and infinity when a given CpG pair is demethylated exclusively, independently, or concordantly, respectively. *Lower* *right*, Genome browser view of part of the *Il4* locus, with amplicons indicated as in **Fig. 1C**. (**B**) Positions of CpGs in the indicated amplicons: *Il4* *CNS1* (CpGs 9-13), *Il4* *CNS1* (CpGs 14-17), *Il4* *CNS2* (CpGs 48-51), promoter, HSII-3’, and Exon 3. (**C**) Methylation profiles for the indicated amplicons in Th2 cells after the first (*upper*) and second (*lower*) cycles of differentiation. Each row represents one read. Black indicates methylation (5mC+5hmC) and white indicates the presence of unmodified C, 5fC or 5caC at the indicated CpG. (**D**) Matrix showing Odds ratio of any two CpGs as a measure of concordant modification in Th2 cells after the first (*upper*) and second (*lower*) cycles of differentiation. The brighter the red colour, the more similar the methylation status of the CpGs being compared. (**E**) For all possible pairs of CpGs, the Odds ratio for a pair of CpGs is plotted against the distance between that pair of CpGs.
