# Supplementary material for: Roles of TET and TDG in DNA demethylation in proliferating and non-proliferating immune cells
Source: Genome Biol. 2021 Jun 22;22:186. doi: 10.1186/s13059-021-02384-1 (PMC8218415; doi:10.1186/s13059-021-02384-1)
Supplement: Supplementary file 5 — Additional file 5. Review history. [file 13059_2021_2384_MOESM5_ESM.docx]

Review History

**First round of review**

**Reviewer 1**

**Are you able to assess all statistics in the manuscript, including the appropriateness of statistical tests used?**

Yes, and I have assessed the statistics in my report.

**Comments to author:**

Tight control of DNA methylation status is essential for numerous cellular process including proliferation, differentiation, and survival. Yet, the relative importance of active (proliferation-independent) verses passive DNA demethylation remains unknown. The study by Onodera and colleagues provides additional insight into this crucial biologic process through the comparison of well controlled genetic models. The authors clearly demonstrate that in type 2 helper T cell differentiation TET-mediated DNA demethylation occurs predominantly in a cell proliferation dependent manner. Specifically, T cells lacking TDG, which can remove 5fC and 5caC independent of proliferation, display no impairment in IL-4 production or T cell development. Additionally, the authors also investigate the role of TDG-mediated DNA demethylation in the non-proliferative process of macrophage activation following LPS exposure. Similarly to T cells, loss of TET-mediated DNA demethylation significantly altered macrophage activation, while loss of TDG resulted in a minimal effect. Combined, these data further support the notion that TDG-mediated DNA demethylation plays a relatively minor role at least in immune cell function. In addition to these well done studies, the paper also notes a few fascinating observations that provide interesting directions for future studies. Overall, the paper is suitable for publication; however, responses to the few comments/questions highlighted below may further improve the manuscript and/or future studies.

Comments:

1. The authors mention in the introduction that alterations in the DNA methylation machinery (DMNT1/Uhrf1) may play a role in demethylation. Are the levels of these proteins decreased following T cell/macrophage activation? Do these levels change in TET deficient and TDG deficient cells?

2. In figure 3 the authors demonstrate that 5hmC is enhanced in macrophages following LPS stimulation, but that this process is independent of TET enzymes? In addition, in figure 1 the authors identify multiple sites in the IL-4 locus that are demethylated following activation, but seem to occur independent of both the TET and TDG activity? What other mechanisms could be involved in this process? Linking back to comment #1, could this be secondary to diminished DNA methylation machinery? Although this does not explain the increase if 5hmC.

3. The authors correctly use BMDMs as a model of a non-proliferative cell type. However, activation of BMDMs with LPS still results in a change in cellular state. Another interesting model system to study this in would be in T cells that have already selected a lineage. This group has done a considerable amount of work on the role TET enzymes in regulatory T cells. Does inducible loss of TETs or TDG following selection of a cell state (stable Foxp3 or GATA3 expression) impair the continued stability and function of these cells? Could active DNA demethylation be important in this context? The authors could try this by giving tamoxifen in culture after polarization to the Th2 or Treg lineage.

**Reviewer 2**

**Are you able to assess all statistics in the manuscript, including the appropriateness of statistical tests used?**

Yes, and I have assessed the statistics in my report.

**Comments to author:**

The manuscript entitled "Roles of TET and TDG in DNA demethylation in proliferating and non-proliferating immune cells" applied the deep DNA modification sequencing methods (BS seq and PB seq) to reveal the TET and TDG's role in differentiating Th2 cells and LPS-induced BMDM cells. This study identifies TETs, but not TDG, whose deficiency will impair DNA demethylation, gene expression (like II4), Th2 and BMDM cell differentiation. This study clearly shows the passive DNA demethylation pathway is a major contribution to DNA demethylation during cell differentiation, while TDG, a thymine DNA glycosylase, indeed excises 5fc and 5cac, but has minimal effect on DNA demethylation, gene expression, and cell differentiation. This study also provides a novel method for exploring concordant demethylation patterns which could benefit later related studies. Overall, this study is convincing and meaningful, which expands our understanding of TET and TDG's role in DNA demethylation regulation in certain biological processes.

Some minor comments:

1, As the author mentioned, upon LPS stimulation, the most highly enriched motif was for NFKB; and GATA3 is also related to TET-dependent II4 gene demethylation. Based on previous reports, could these two TFs recruit TET proteins and mediate the DNA demethylation?

2, The author mentioned DNA demethylation is a consequence, not a cause, of gene expression. On the other hand, many studies showed TETs could activate gene expression by DNA demethylation. The author could give more thoughts on this different opinion in their discussion.

**Authors Response**

**Point-by-point responses to the reviewers’ comments:**

Comment

Reviewer #1:

Tight control of DNA methylation status is essential for numerous cellular process including proliferation, differentiation, and survival. Yet, the relative importance of active (proliferation-independent) verses passive DNA demethylation remains unknown. The study by Onodera and colleagues provides additional insight into this crucial biologic process through the comparison of well controlled genetic models. The authors clearly demonstrate that in type 2 helper T cell differentiation TET-mediated DNA demethylation occurs predominantly in a cell proliferation dependent manner. Specifically, T cells lacking TDG, which can remove 5fC and 5caC independent of proliferation, display no impairment in IL-4 production or T cell development. Additionally, the authors also investigate the role of TDG-mediated DNA demethylation in the non-proliferative process of macrophage activation following LPS exposure. Similarly to T cells, loss of TET-mediated DNA demethylation significantly altered macrophage activation, while loss of TDG resulted in a minimal effect. Combined, these data further support the notion that TDG-mediated DNA demethylation plays a relatively minor role at least in immune cell function. In addition to these well-done studies, the paper also notes a few fascinating observations that provide interesting directions for future studies. Overall, the paper is suitable for publication; however, responses to the few comments/questions highlighted below may further improve the manuscript and/or future studies.

Comments:

1. The authors mention in the introduction that alterations in the DNA methylation machinery (DMNT1/Uhrf1) may play a role in demethylation. Are the levels of these proteins decreased following T cell/macrophage activation? Do these levels change in TET deficient and TDG deficient cells?

*Response: TET iTKO Th2 cells: Published data for naïve CD4 T cells under-going Th1 and Th2 differentiation (GSE159505) showed that the levels of Dnmt1 and Uhrf1 transcripts increased about 5-fold during the course of Th1/Th2 differentiation, peaking at 4 days after activa-tion, and declining somewhat thereafter (Figure 1 for reviewer). Based on these data, we performed western blotting for DNMT1 and UHRF1 proteins at day 6 of Th2 differentiation; the DNMT1 antibody has been validated in our lab as specific for DNMT1 using mouse embryonic stem cells deficient for all three DNMTs, while the specifi-city of the UHRF1 antibody has been validated by the manufacturer. Although Western blotting is not a quantitative technique, we observed a clear decrease in both DNMT1 and UHRF1 protein levels in TET iTKO compared to WT Th2 cells, without any perceptible change in either DNMT1 or UHRF1 protein levels in WT compared to Tdg iKO Th2 cells (new Figs. S1P and S1Q). Macrophages. DNMT1. There was a slight increase (estimated ~2- to 3-fold) in DNMT1 protein in prolife-rating TET iTKO and Tdg iKO BMDMs compared to WT BMDMs; these levels decreased following LPS stimulation in all three cell types, but the difference between cell types was maintained (Figure 2B for reviewer, top panels). UHRF1. There was a slight increase (estimated ~2-fold) in UHRF1 protein in TET iTKO compared to WT cells; these levels decreased somewhat following LPS stimulation but the difference between WT and TET iTKO cells was maintained (Figure 2B for reviewer, left middle panel). UHRF1 expression in macrophages was low and hard to detect by Western blotting, and so was not monitored in Tdg iKO cells. Summary. Overall, the observed differences in DNMT1 and UHRF1 expression may partly account for the differences in Th2 differentiation and macrophage activation between WT and TET iTKO cells. We have added a sentence to explain this effect in the revised manuscript (please see the bottom of page 6).*

2. In figure 3 the authors demonstrate that 5hmC is enhanced in macrophages following LPS stimulation, but that this process is independent of TET enzymes?

*Response: We apologize for the confusion, and have clarified these points in the revised version of the manuscript. Fig. 3B-D and 3F show data from wildtype macrophages only, either unstimulated or stimulated with LPS. Fig. 3B shows that ~4000 of a total of ~136,000 enhancers in wild type macrophages gain 5hmC and an equal number lose 5hmC after LPS stimulation; while Fig. 3C shows an example of an enhancer located 5’ of the Batf gene that gains 5hmC in wild type macrophages during the 6 hours of LPS stimulation. For regions showing an increase in 5hmC, Fig. 3D shows – again in wild type macrophages – the increase in 5hmC plotted against the change in expression of the associated gene (nearest TSS defined using HOMER “annotatePeaks.pl” function). The reviewer may have been confused by the data in Fig. 3E, in which the expression of selected genes in WT, TET iTKO and TDG iKO cells are compared before and after 6 hours of LPS stimulation. We have changed the y-axis of this figure to make it clear that we are plotting normalized read counts from RNA-seq.*

In addition, in figure 1 the authors identify multiple sites in the IL-4 locus that are demethylated following activation, but seem to occur independent of both the TET and TDG activity? What other mechanisms could be involved in this process? Linking back to comment #1, could this be secondary to diminished DNA methylation machinery? Although this does not explain the increase of 5hmC.

*Response: The reviewer is referring to 5 CpGs in the region that we have labelled “DNase HSII 3’ region” in Fig. 1C (indicated with a blue bar below the Il4 gene in Figs. 1C and 1D). These 5 CpGs are demethylated relative to naïve T cells in WT Th2 cells (Fig. 1D, compare black and grey bars); unlike CpGs in the other enhancer regions indicated, they continue to be demethylated in TET iTKO as well as Tdg iKO Th2 cells (Fig. 1D), and do not gain 5fC and 5caC in Tdg iKO Th2 cells (Fig. 1E).*

*The mechanism of demethylation of this region is a very interesting question that we are unfortunately not yet able to answer, but can only speculate about. It seems unlikely that the estimated ~2-fold decrease in DNMT1 expression in TET iTKO compared to WT Th2 cells accounts for the apparent TET-independent demethylation. Possibilities are that (i) there is a TET-independent mechanism of DNA demethylation that operates only in certain regions of the genome; or (ii) the maintenance DNA methyltransferase DNMT1 is somehow excluded from the DNase HSII 3’ region, possibly because of the adjacent annotated noncoding RNA, NR_027491 (shown as a blue bar within intron 2 of the Il4 gene) is selectively transcribed in Th2 cells compared to naïve T cells. Experiments to address these possibilities are underway. We have added a paragraph discussing this point to the revised manuscript (please see pages 12-13).*

3. The authors correctly use BMDMs as a model of a non-proliferative cell type. However, activation of BMDMs with LPS still results in a change in cellular state. Another interesting model system to study this in would be in T cells that have already selected a lineage. This group has done a considerable amount of work on the role TET enzymes in regulatory T cells. Does inducible loss of TETs or TDG following selection of a cell state (stable Foxp3 or GATA3 expression) impair the continued stability and function of these cells? Could active DNA demethylation be important in this context? The authors could try this by giving tamoxifen in culture after polarization to the Th2 or Treg lineage.

*Response: To address this comment, we assessed whether inducible loss of TETs or TDG could affect GATA3 protein expression and/or stability by first polarizing the cells to the Th2 lineage and then treating the differentiated Th2 cells with 4-hydroxytamoxifen (4-HT) (new Fig. S1G and S1H). We found that GATA3 protein expression levels were not affected by TET or TDG deletion. We also examined Foxp3 stability in restimulated iTreg cells that were induced by activation in the presence of IL-2, TGF-, retinoic acid and vitamin C (new Fig. S1L and S1M), the optimal conditions for stable Foxp3 expression by the differentiated iTreg cells. Under these optimal conditions, Foxp3 protein stability was not affected by TDG deletion. Thus, expression of both Foxp3 and IL-4, by differentiating iTregs and Th2 cells respectively, requires TET but not TDG. We have added a few sentences to explain this in the revised manuscript (please see pages 4-5).*

Reviewer #2:

The manuscript entitled "Roles of TET and TDG in DNA demethylation in proliferating and non-proliferating immune cells" applied the deep DNA modification sequencing methods (BS seq and PB seq) to reveal the TET and TDG's role in differentiating Th2 cells and LPS-induced BMDM cells. This study identifies TETs, but not TDG, whose deficiency will impair DNA demethylation, gene expression (like II4), Th2 and BMDM cell differentiation. This study clearly shows the passive DNA demethylation pathway is a major contribution to DNA demethylation during cell differentiation, while TDG, a thymine DNA glycosylase, indeed excises 5fc and 5cac, but has minimal effect on DNA demethylation, gene expression, and cell differentiation. This study also provides a novel method for exploring concordant demethylation patterns which could benefit later related studies. Overall, this study is convincing and meaningful, which expands our understanding of TET and TDG's role in DNA demethylation regulation in certain biological processes.

Some minor comments:

1. As the author mentioned, upon LPS stimulation, the most highly enriched motif was for NFkB; and GATA3 is also related to TET-dependent II4 gene demethylation. Based on previous reports, could these two TFs recruit TET proteins and mediate the DNA demethylation?

*Response: Several transcription factors, including PU.1 and E2A (PMID 27869616), have been shown to bind TET2 through a protein-protein interaction that persists in the absence of DNA (i.e. after treatment with benzonase, a nonspecific RNA and DNA nuclease). For instance, in osteoclasts, transcription factor binding motif analysis revealed an over-representation of PU.1, NF-κB, and AP-1 (Jun/Fos) binding motifs in genes undergoing DNA methylation changes (PMID: 24028770); and PU.1 but not NF-B (p65) was shown to interact with TET2 in a co-IP experiment. However, when we tested for co-IP between TET2 and GATA3 (both overexpressed in HEK293 cells) in the presence of benzonase in whole cell lysates, we did not observe any co-immunoprecipitation (not shown). This result suggests that if TET2 recruits GATA3 in vivo, the interaction is of low affinity and precludes efficient pulldown under our buffer conditions. Alternatively, the interaction could be mediated indirectly through intermediary proteins or nucleic acids within a larger complex. We have added a few sentences of further explanation to the discussion (please see the middle of page 12).*

2. The author mentioned DNA demethylation is a consequence, not a cause, of gene expression. On the other hand, many studies showed TETs could activate gene expression by DNA demethylation. The author could give more thoughts on this different opinion in their discussion.

*Response: The conclusion is based on the observations by many groups that even if DNA at a regulatory element (enhancer) is demethylated, expression of the associated gene(s) requires other factors, including the binding of transcription factors which recruit histone acetyltransferases and chromatin remodeling complexes to the regulatory elements. The case is made clearly in two outstanding reviews that are cited in our manuscript (references 29 and 30). We have added a few sentences of further explanation to the discussion (please see the bottom of page 13).*

**Second round of review**

**Reviewer 1**

This is an excellent paper and the revisions have only improved it. Job well done!
